# Supplementary material for: Accurate atomic resolution XFEL structures of a metalloenzyme reveal key insights into its catalytic mechanism*
Source: Nat Commun. 2026 Mar 7;17:3735. doi: 10.1038/s41467-026-70261-1 (PMC13103080; doi:10.1038/s41467-026-70261-1)
Supplement: Supplementary file 1 — Supplementary Information [file 41467_2026_70261_MOESM1_ESM.pdf]

SUPPLEMENTARY INFORMATION

Accurate atomic resolution XFEL structures of a metalloenzyme reveal key insights into its catalytic mechanism

Samuel L. Rose[+1†], Svetlana Antonyuk[+1], Felix F. Ferroni[2], Hiroshi Sugimoto[3], Keitaro Yamashita[4], Kunio Hirata[3], Hideo Ago[3], Go Ueno[3], Hironori Murakami[3], Robert. R. Eady[1], Takehiko Tosha[*5], Masaki Yamamoto[*4], S. Samar Hasnain[*1]

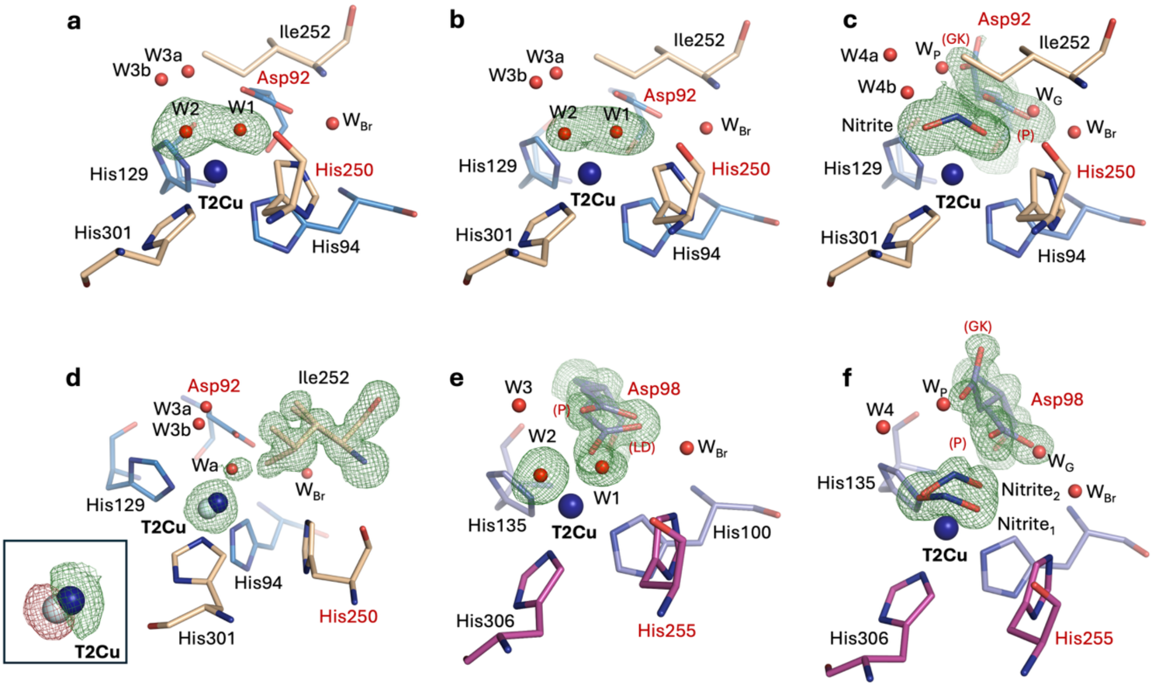

Supplementary Fig 1. F_o-F_c omit maps of T2Cu ligands and alternative conformations in the atomic resolution XFEL structures

(**a**) The F_o-F_c omit map for the atomic resolution (1.15 Å) XFEL structure of oxidised as-isolated *Br*NiR at pH 5.5 showing positive density for the two T2Cu solvent ligands. (**b**) The F_o-F_c omit map for the atomic resolution (1.00 Å) XFEL structure of oxidised as-isolated *Br*NiR at pH 7.3 showing density for the two T2Cu solvent ligands. (**c**) The F_o-F_c omit map for the atomic resolution (1.02 Å) XFEL structure of oxidised nitrite-bound *Br*NiR showing positive density for the single T2Cu nitrite molecule coordinated in "top-hat" binding mode and the F_o-F_c density around the catalytic Asp residue showing positive density for both the proximal and gatekeeper conformations of the residue. (**d**) The F_o-F_c omit map for the atomic resolution (1.05 Å) XFEL structure of dithionite-reduced *Br*NiR at pH 5.5 showing positive density for dual positions of the T2Cu and a single T2Cu solvent ligand. The F_o-F_c density around the catalytic Ile residue showing positive density for the flipping of CD1 side-chain into the active site pocket. The inset panel shows the F_o-F_c density if only one T2Cu ion is modelled – negative density (-3σ) is observed around the single position of T2Cu and positive density (+3σ) is observed around the second additional position of the T2Cu. (**e**) The F_o-F_c omit map for the sub-atomic resolution (0.95 Å) XFEL structure of oxidised as-isolated *Ac*NiR at pH 4.8 showing positive density for the two T2Cu solvent ligands and the F_o-F_c density around the catalytic Asp residue showing positive density for both the proximal and lower-distorted conformations of the residue. (**f**) The F_o-F_c omit map for the sub-atomic resolution (0.95 Å) XFEL structure of oxidised nitrite-bound *Ac*NiR at pH 4.8 showing positive density for two positions of the T2Cu nitrite molecule coordinated in "top-hat" binding modes and the F_o-F_c density around the catalytic Asp residue showing positive density for both the proximal and gatekeeper conformations of the residue. For *Br*NiR structures, residues from chain A are coloured in blue and chain B residues are coloured in gold and for *Ac*NiR structures, residues from chain A are coloured in purple and chain B residues are coloured magenta. Residues are labelled in black and catalytic Asp and His residues are labelled in red. Waters are coloured in red. P = proximal Asp_{CAT} conformation, GK = gatekeeper Asp_{CAT} conformation, LD = lower distorted Asp_{CAT} conformation, W_{Br} = bridging water, W_{GK} = gatekeeper water, W_P = proximal water. All F_o-F_c omit maps are contoured to 3σ and coloured in green.

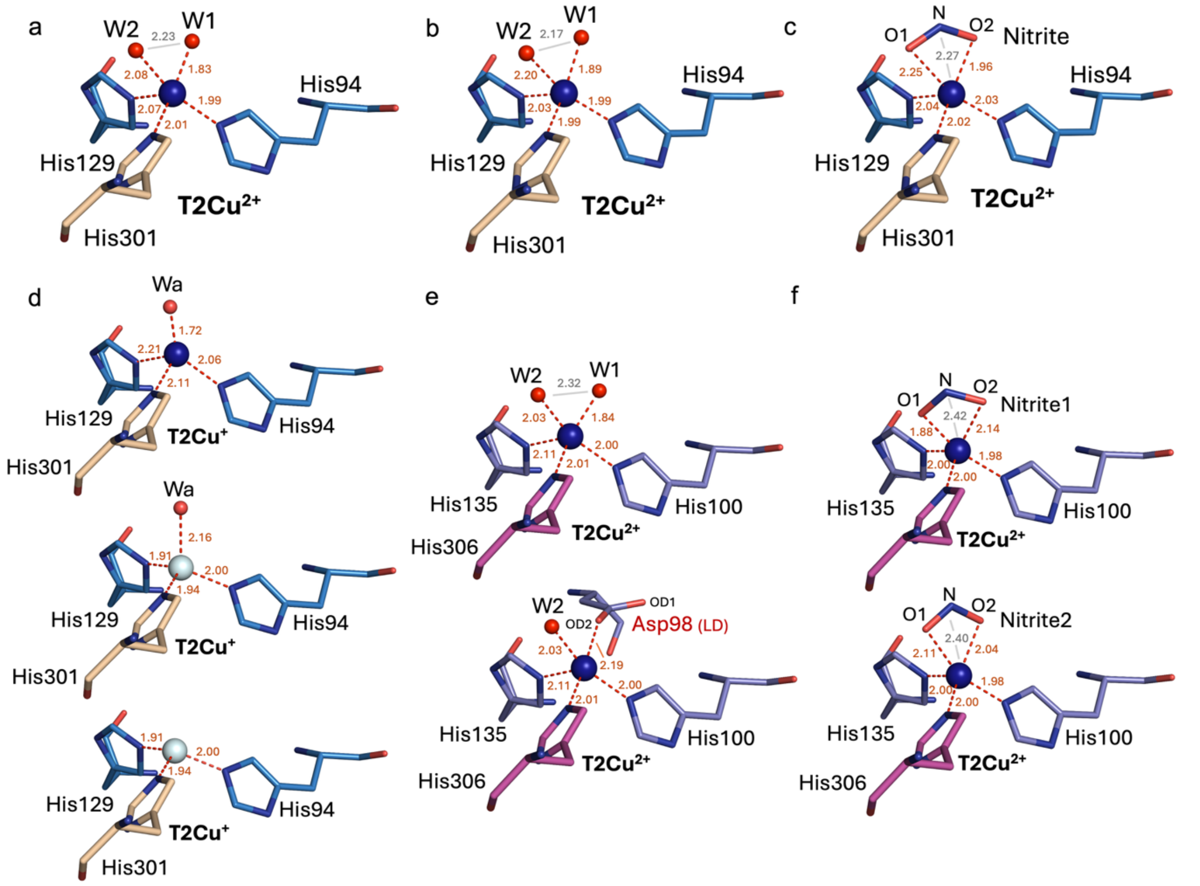

Supplementary Fig 2. T2Cu coordination sites in the atomic resolution XFEL structures after unrestrained SHELXL refinement

(**a**) The pentacoordinated T2Cu(II) coordination site in the atomic resolution (1.15 Å) XFEL structure of oxidised as-isolated *Br*NiR at pH 5.5. (**b**) The pentacoordinated T2Cu(II) coordination site in the atomic resolution (1.00 Å) XFEL structure of oxidised as-isolated *Br*NiR at pH 7.3. (**c**) The T2Cu(II) coordination site in the atomic resolution (1.02 Å) XFEL structure of oxidised nitrite-bound *Br*NiR. (**d**) The T2Cu(I) coordination site in the atomic resolution (1.05 Å) XFEL structure of dithionite-reduced *Br*NiR at pH 5.5. Three T2Cu(I) coordination conformations are shown from top to bottom: Tetracoordinated T2Cu(I) with single solvent ligand, tetracoordinated T2Cu(I) with single solvent ligand/ T2Cu(I) dropping into the histidine plane and tri-coordinated T2Cu(I). (**e**) The pentacoordinated T2Cu(II) site in the sub-atomic resolution (0.95 Å) XFEL structure of oxidised as-isolated *Ac*NiR at pH 4.8. Two T2Cu(II) coordination conformations are shown from top to bottom: pentacoordinated T2Cu(II) site with two solvent ligands and pentacoordinated T2Cu(II) site with a solvent ligand and direct coordination from Asp_{CAT}. (**f**) The pentacoordinated T2Cu(II) site in the sub-atomic resolution (0.95 Å) XFEL structure of oxidised nitrite-bound *Ac*NiR at pH 4.8. Two T2Cu(II) coordination conformations are shown from top to bottom: pentacoordinated T2Cu(II) with nitrite in position 1 and pentacoordinated T2Cu(II) with nitrite in position 2 – both have bidentate coordination via the two oxygen atoms in a "top-hat" binding mode. For *Br*NiR structures, residues from chain A are coloured in blue and chain B residues are coloured in gold and for *Ac*NiR structures, residues from chain A are coloured in purple and chain B residues are coloured magenta. Residues are labelled in black and catalytic Asp and His residues are labelled in red. Waters are coloured in red. LD = lower distorted Asp_{CAT} conformation, W_{Br} = bridging water. Coordination distances in red are in Å. All models are after unrestrained refinement in SHELXL.

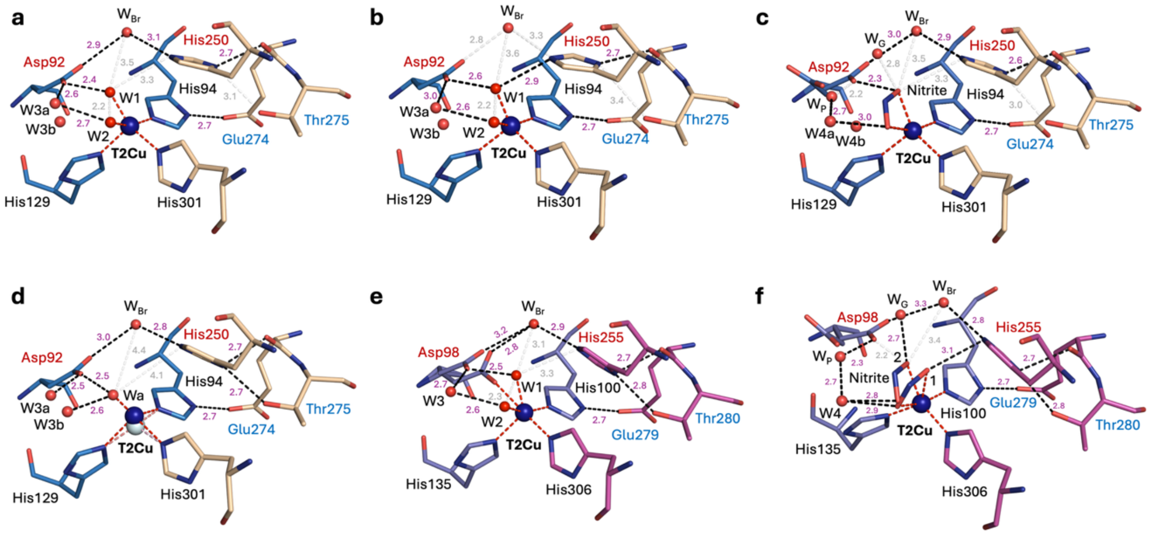

Supplementary Fig 3. Extended T2Cu active site in the atomic resolution XFEL structures and bond distances

(**a**) The extended T2Cu active site for the atomic resolution (1.15 Å) XFEL structure of oxidised as-isolated *Br*NiR at pH 5.5 revealing bond distances around the site and bonding distances of the catalytic His reside (His$_{CAT}$) to local residues Thr/Glu. (**b**) The extended T2Cu active site for the atomic resolution (1.00 Å) XFEL structure of oxidised as-isolated *Br*NiR at pH 7.3 revealing bond distances around the site and bonding distances of the catalytic His reside (His$_{CAT}$) to local residues Thr/Glu. (**c**) The extended T2Cu active site for the atomic resolution (1.02 Å) XFEL structure of oxidised nitrite-bound *Br*NiR at pH 5.5 revealing bond distances around the site and bonding distances of the catalytic His reside (His$_{CAT}$) to local residues Thr/Glu. (**d**) The extended T2Cu active site for the atomic resolution (1.05 Å) XFEL structure of dithionite-reduced as-isolated *Br*NiR at pH 5.5 revealing bond distances around the site and bonding distances of the catalytic His reside (His$_{CAT}$) to local residues Thr/Glu. (**e**) The extended T2Cu active site for the atomic resolution (0.95 Å) XFEL structure of oxidised as-isolated *Ac*NiR at pH 4.8 revealing bond distances around the site and bonding distances of the catalytic His reside (His$_{CAT}$) to local residues Thr/Glu. (**f**) The extended T2Cu active site for the atomic resolution (0.95 Å) XFEL structure of oxidised nitrite-bound *Ac*NiR at pH 4.8 revealing bond distances around the site and bonding distances of the catalytic His reside (His$_{CAT}$) to local residues Thr/Glu. For *Br*NiR structures, residues from chain A are coloured in blue and chain B residues are coloured in gold and for *Ac*NiR structures, residues from chain A are coloured in purple and chain B residues are coloured magenta. Residues are labelled in black, catalytic Asp and His residues are labelled in red and the Thr and Glu residues are labelled in blue. Waters are coloured in red. P = proximal Asp$_{CAT}$ conformation, GK = gatekeeper Asp$_{CAT}$ conformation, LD = lower distorted Asp$_{CAT}$ conformation, W$_{Br}$ = bridging water, W$_{GK}$ = gatekeeper water, W$_P$ = proximal water. Red dashed lines indicate T2Cu coordination, black dashed lines indicate likely bonding, grey dashed lines indicate unlikely bonding. Bond distances are in Å. All models are after unrestrained refinement in SHELXL.

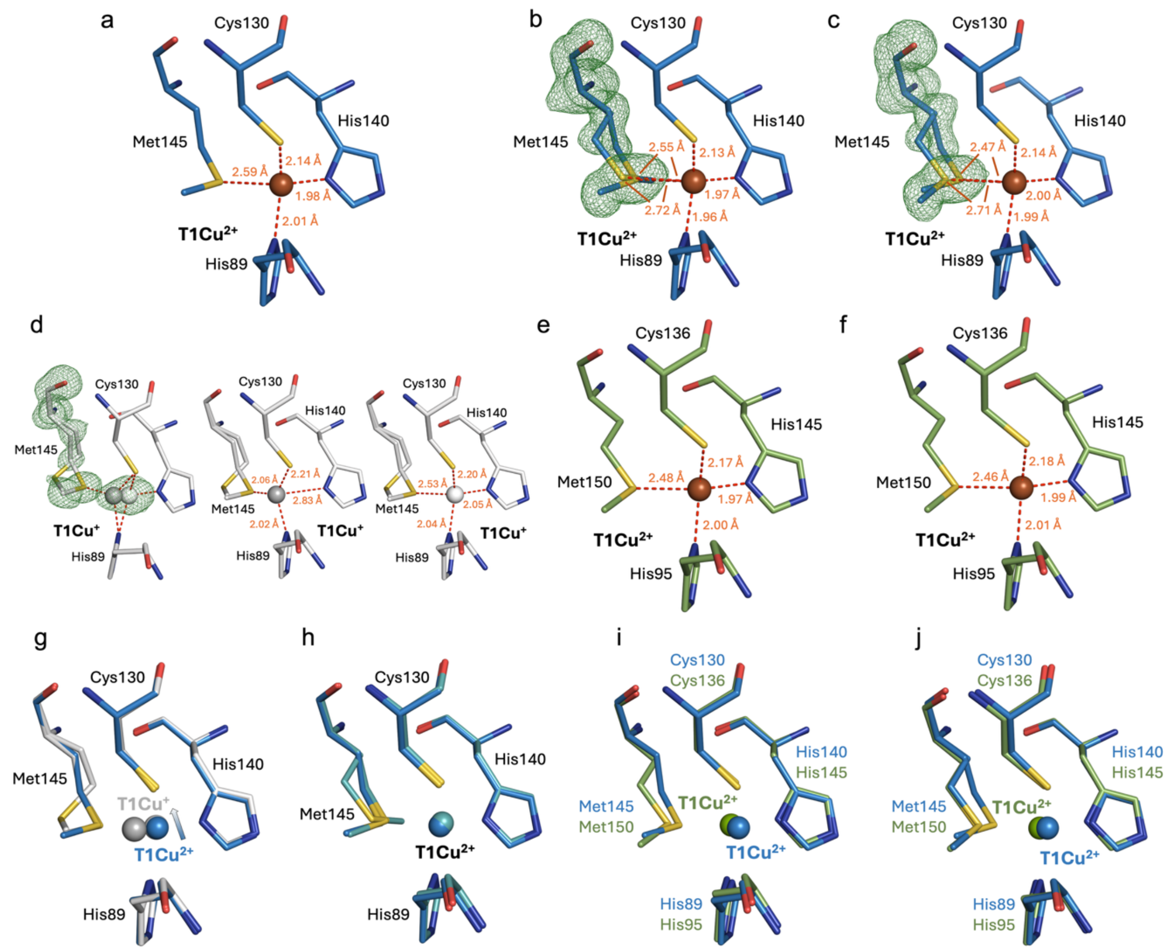

Supplementary Fig 4. T1Cu coordination sites in the atomic resolution XFEL structures of bluish-green and green CuNiRs

(**a**) The tetracoordinated T1Cu(II) coordination site in the atomic resolution (1.15 Å) XFEL structure of oxidised as-isolated bluish-green *Br*NiR at pH 5.5 (blue). (**b**) The tetracoordinated T1Cu(II) coordination site for the atomic resolution (1.00 Å) XFEL structure of oxidised as-isolated bluish-green *Br*NiR at pH 7.3 (blue). F_o-F_c omit map is shown around the T1Cu coordinating Met residue showing positive density for two unique positions. (**c**) The tetracoordinated T1Cu(II) coordination site for the atomic resolution (1.02 Å) XFEL structure of oxidised nitrite-bound bluish-green *Br*NiR at pH 7.3 (blue). F_o-F_c omit map is shown around the T1Cu coordinating Met residue showing positive density for two unique positions (**d**) The T1Cu(I) coordination site for the atomic resolution (1.05 Å) XFEL structure of dithionite-reduced colourless *Br*NiR at pH 5.5 (white). F_o-F_c omit map is shown around the T1Cu coordinating Met residue and the T1Cu ion showing positive density for two unique positions of each. (**e**) The tetracoordinated T1Cu(II) site for the sub-atomic resolution (0.95 Å) XFEL structure of oxidised as-isolated green *Ac*NiR at pH 4.8 (green). (**f**) The tetracoordinated T1Cu(II) site for the sub-atomic resolution (0.95 Å) XFEL structure of oxidised nitrite-bound green *Ac*NiR at pH 4.8 (green). (**g**) Comparison between the oxidised T1Cu(II) (blue) and reduced T1Cu(I) (white) coordination sites of *Br*NiR atomic resolution XFEL structures. (**h**) Comparison between the oxidised T1Cu(II) coordination sites of *Br*NiR at low (blue) and high pH (teal) for their respective atomic resolution XFEL structures. (**i**) Comparison between the as-isolated oxidised T1Cu(II) coordination sites of bluish-green *Br*NiR (blue) and green *Ac*NiR (green) in their atomic resolution XFEL structures. (**j**) Comparison between the nitrite-bound oxidised T1Cu(II) coordination sites of bluish-green *Br*NiR (blue) and green *Ac*NiR (green) in their atomic resolution XFEL structures. Residues are labelled in black and coordination distances are shown as dashed red lines. Coordination distances in orange are in Å. All models are after unrestrained refinement in SHELXL. F_o-F_c omit maps are contoured to 3σ and coloured in green.

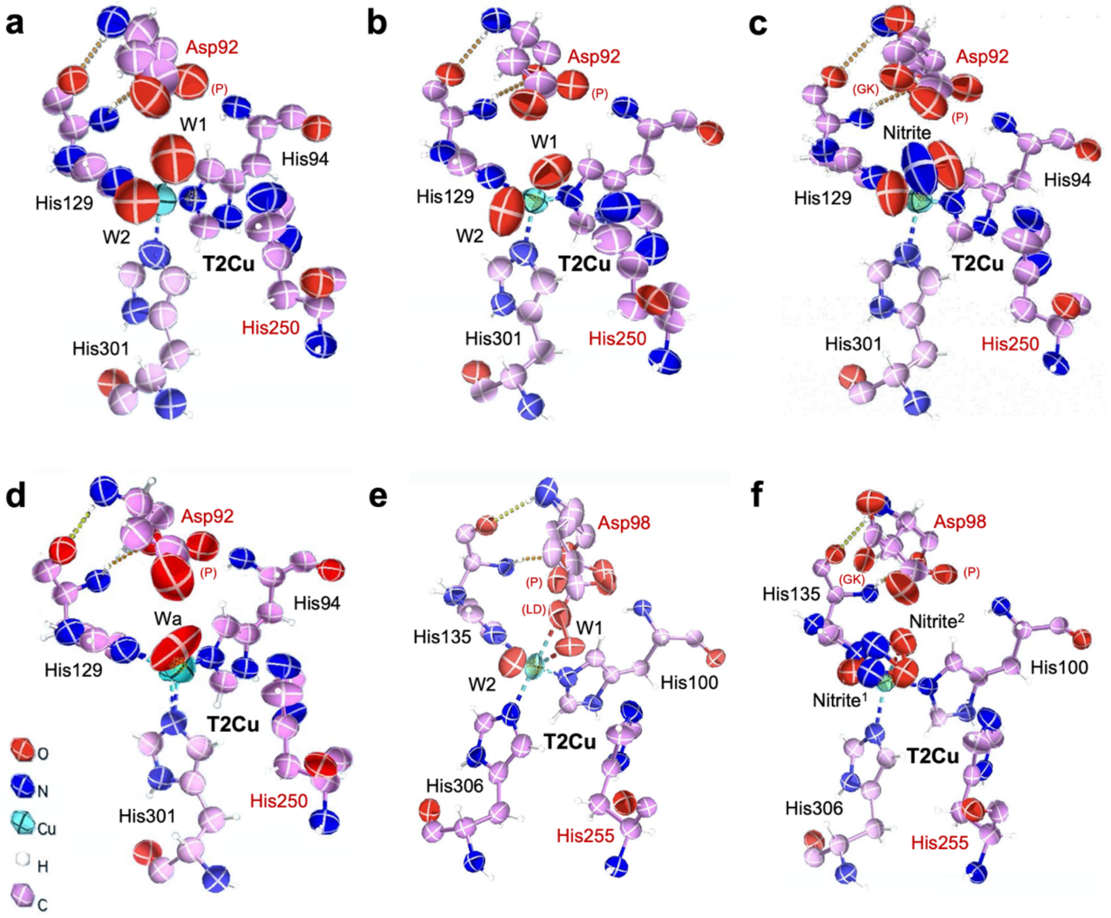

Supplementary Fig 5. Ellipsoidal models of the T2Cu active sites in the atomic resolution macromolecular XFEL structures

(**a**) Ellipsoidal model of the T2Cu site in the atomic resolution (1.15 Å) XFEL structure of as-isolated *Br*NiR at pH 5.5, showing the anisotropic displacement parameters (ADPs) with 50 % probability. (**b**) Ellipsoidal model of the T2Cu site in the atomic resolution (1.00 Å) XFEL structure of as-isolated *Br*NiR at pH 7.3, showing the ADPs with 50 % probability. (**c**) Ellipsoidal model of the T2Cu site in the atomic resolution (1.02 Å) XFEL structure of nitrite-bound *Br*NiR at pH 5.5, showing the T1Cu and T2Cu sites, showing the ADPs with 50 % probability (**d**) Ellipsoidal model of the T2Cu site in the atomic resolution (1.05 Å) XFEL structure of dithionite-reduced *Br*NiR at pH 5.5, showing the ADPs with 50 % probability. (**e**) Ellipsoidal model of the T2Cu site in the atomic resolution (0.95 Å) XFEL structure of as-isolated *Ac*NiR at pH 4.8, showing the ADPs with 50 % probability. (**f**) Ellipsoidal model of the T2Cu site in the atomic resolution (0.95 Å) XFEL structure of nitrite-bound *Ac*NiR at pH 4.8, showing the ADPs with 50 % probability. P = proximal Asp_{CAT} conformation, GK = gatekeeper Asp_{CAT} conformation, LD = lower distorted Asp_{CAT} conformation

Supplementary Table 1. Details of the structures obtained using the higher energy X-ray free electron laser (XFEL) serial femtosecond rotational crystallography (SF-ROX) collection method at 13 keV and the atomic resolutions achieved.

Enzyme	No. of crystals	Average crystal size	Resolution
Br^{2D}NiR (pH 5.5) Oxidised	42	600 µm^3	1.10 Å
*BrJ*NiR (pH 7.3) Oxidised	64	600 µm^3	1.00 Å
*BrJ*NiR + NO_2^- (pH 5.5) Oxidised	64	600 µm^3	1.02 Å
Br^{2D}NiR + $[S_2O_4]^{2-}$ (pH 5.5) Reduced	64	450 µm^3	1.05 Å
*Ac*NiR (pH 4.8) Oxidised	48	450 µm^3	0.95 Å
*Ac*NiR + NO_2^- (pH 4.8) Oxidised	48	450 µm^3	0.95 Å

Supplementary Table 2. **Data collection and refinement statistics.**

	*Br*NiR as isolated (pH 5.5)	*Br*NiR as isolated (pH 7.3)	*Br*NiR nitrite-bound (pH 5.5)	*Br*NiR chemically reduced (pH 5.5)	*Ac*NiR as isolated (pH 4.8)	*Ac*NiR nitrite-bound (pH 4.8)
**Data collection**						
Space group	$P2_13$	$P6_3$	$P6_3$	$P2_13$	$P2_13$	$P2_13$
Images merged	2526	4151	3077	1499	1806	1032
Cell dimensions						
a=b=c; a=b,c (Å)	107.32	103.82, 64.47	103.89, 64.35	106.99	95.25	95.32
Resolution (Å)	17.00-1.10 (1.11-1.10) *	17.00-1.00 (1.01-1.00) *	17.00-1.00 (1.01-1.00) *	17.00-1.05 (1.06-1.05) *	17.00-0.95 (0.96-0.95) *	17.00-0.95 (0.96-0.95) *
R_{split} (%)	0.122 (0.964)	0.160 (1.054)	0.166 (0.986)	0.228 (0.841)	0.187 (0.860)	0.238 (1.048)
$CC_{½}$	0.975 (0.225)	0.935 (0.202)	0.935 (0.206)	0.890 (0.262)	0.927 (0.336)	0.853 (0.235)
$I / \sigma I$	6.5 (1.4)	6.1 (1.2)	5.3 (1.2)	3.8 (1.4)	4.8 (1.3)	4.0 (1.2)
Completeness (%)	99.8 (94.7)	100 (99.2)	99.99 (99.96)	99.9 (100)	99.9 (100)	99.9 (99.8)
Redundancy	157.9 (10.0)	104.0 (18.1)	85.33 (30.8)	61.7 (42.2)	100.4 (21.3)	58.0 (15.2)
Wilson B ($Å^2$)	10.5	8.8	8.8	8.8	6.3	6.2
**Refinement**						
Resolution (Å)	17.00-1.15	17.00-1.00	17.00-1.02	17.00-1.05	17.00-0.95	17.00-0.95
No. reflections	165,516	212,579	212,451	218,286	179,931	180,355
R_{work} / R_{free}	0.150/0.174	0.134/0.157	0.139/0.169	0.148/0.168	0.134/0.161	0.145/0.170
No. atoms						
Protein	2750	2682	2651	2663	2661	2685
Ligand/ion	92/2	43/2	55/2	104/2	20/2	14/2
Water	444	507	484	420	511	513
B-factors ($Å^2$)						
Protein	16.17	13.56	13.25	12.86	8.50	8.30
Ligand/Cu	40.49/12.58	25.92/9.89	33.00/9.77	28.16/9.52	21.02/6.40	18.8/5.9
Water	32.67	31.20	30.48	30.46	24.90	23.30
R.m.s. deviations						
Bond lengths (Å)	0.016	0.016	0.015	0.015	0.014	0.016
Bond angles (°)	1.99	1.92	1.94	1.94	1.91	2.10
**PDB ID**	**9ROS**	**9RO1**	**9RNZ**	**9ROC**	**9RLL**	**9RN0**

Number of crystals used for SF-ROX data collection – *Br*NiR as isolated - pH 5.5 (**41**); *Br*NiR as isolated - pH 7.3 (**64**); *Br*NiR nitrite-bound (**48**); *Br*NiR chemically reduced (**64**); *Ac*NiR as isolated (**48**); *Ac*NiR nitrite-bound (**48**)

* values in parentheses are for highest-resolution shell

Supplementary Table 3. Distances between T1Cu and T2Cu to their ligands.

	***Br*NiR as-isolated (pH 5.5)**	***Br*NiR as-isolated (pH 7.3)**	***Br*NiR nitrite-bound (pH 5.5)**	***Br*NiR dithionite-reduced (pH 5.5)**	***Ac*NiR as-isolated (pH 4.8)**	***Ac*NiR nitrite-bound (pH 4.8)**
**T1Cu**						
His89(95) $N^{\delta1}$	2.00 (1)	1.96 (1)	1.99 (1)	2.04 (1)/ 2.03 (2)*	2.00 (9)	2.01 (1)
Cys130(136) S^{γ}	2.14 (1)	2.132 (4)	2.14 (4)	2.198 (5)/ 2.21 (2)*	2.17 (4)	2.18 (4)
His140(145) $N^{\delta1}$	1.98 (2)	1.97 (9)	2.00 (1)	2.05 (1)	1.97 (1)	1.99 (1)
Met145(150) S^{δ}	2.59 (1)	2.55 (1)/ 2.72 (1)	2.47 (7) 2.71 (8)	2.53 (1)	2.48 (4)	2.46 (4)
**T2Cu**						
His129(135) $N^{\varepsilon2}$	2.07 (2)	2.03 (1)	2.03 (1)	1.91 (1)/2.21 (1)	2.12 (9)	2.00 (9)
His301(306) $N^{\varepsilon2}$	2.01 (1)	1.99 (1)	2.02 (1)	1.94 (1)/2.11 (1)*	2.01 (9)	2.01 (9)
His94(100) $N^{\varepsilon2}$	1.99 (1)	1.99 (1)	2.02 (1)	2.00 (1) /2.06 (1)*	2.01 (8)	1.98 (9)
Asp98 $O^{\delta2}$ (A)					2.20 (1)	
W1/Wa	1.83 (2)	1.90 (1)		1.72/2.16 (2)*	1.84 (2) (B)	
W2	2.08 (2)	2.20 (2)			2.03 (1)	
NO_2 O^1			1.96 (2)			1.88 (2)/ 2.11 (2)
NO_2 O^2			2.25 (1)			2.14 (2)/ 2.04 (1)
NO_2 N			2.27 (3)			2.42 (3)/ 2.40 (2)

*Distances for T1Cu b or T2Cu b

Supplementary Table 4. Bond lengths and angles of Asp_{CAT} and His_{CAT} in *Br*NiR and AcNiR structures.

Distances (Å) and Angles (°)	***Br*NiR as-isolated (pH 5.5)**	***Br*NiR as-isolated (pH 7.3)**	***Br*NiR nitrite-bound (pH 5.5)**	***Br*NiR chemically reduced (pH 5.5)**	***Ac*NiR as-isolated (pH 4.8)**	***Ac*NiR nitrite-bound (pH 4.8)**
**Resolution (Å)**	**1.15**	**1.00**	**1.02**	**1.05**	**0.95**	**0.95**
**T2Cu occupancy**	**1.0**	**1.0**	**1.0**	**0.67(A)/0.33(B)**	**1.0**	**1.0**
**Asp_{CAT} 92 (98)**						
Occupancy	1.00	1.00	0.17(A)/ 0.83(B) resting	1.00	0.65 (A)/ 0.35 (B) resting	0.32 (A)/ 0.68 (B) resting
C^{γ}-$O^{\delta1}$ (Å)	1.25 (2)	1.23 (2)	1.24 (1)/ 1.26 (2)	1.23 (3)	1.24 (3)/ 1.24 (3)	1.24 (4)/ 1.24 (4)
C^{γ}-$O^{\delta2}$ (Å)	1.27 (2)	1.25 (1)	1.25 (9)/ 1.26 (2)	1.28 (2)	1.34 (4)/ 1.26 (2)	1.25 (2)/ 1.25 (2)
**His_{CAT}250 (255)**						
Occupancy	1.00	1.00	1.00	1.00	1.00	1.00
$C^{\varepsilon1}$-$N^{\varepsilon2}$-$C^{\delta2}$ (°)	108 (2)	107 (2)	109 (2)	108 (1)	108 (1)	108 (1)
C^{γ}-$N^{\delta1}$-$C^{\varepsilon1}$ (°)	113 (2)	108 (2)	115 (2)	110 (1)	111 (1)	109 (1)

Supplementary Table 5. Summary of the protonation states for catalytic residues.

	***Br*NiR as-isolated (pH 5.5)**	***Br*NiR as-isolated (pH 7.3)**	***Br*NiR nitrite-bound (pH 5.5)**	***Br*NiR dithionite-reduced (pH 5.5)**	***Ac*NiR as-isolated (pH 4.8)**	***Ac*NiR nitrite-bound (pH 4.8)**
**Asp_{CAT}**	$O^{\delta2}$ protonation	Non protonated	Non protonated	$O^{\delta2}$ protonation	$O^{\delta2}$ protonation in distorted state	Non protonated
**His_{CAT}**	Fully protonated	Fully protonated	Fully protonated	Fully protonated	Fully protonated	Fully protonated
